# A Criticism of Homœopathy

**Published:** 1909-05-22

**Authors:** C. E. Wheeler


### "A CRITICISM OF HOMŒOPATHY."

*To the Editor of* The Hospital.

Sir,—Despite Dr. Heaney's courtesy I have evidently failed in my previous letter to make the position of homœopathists clear. May I try again?

The believer in homœopathy maintains that, given a case of disease presenting certain symptoms, the remedy most likely to benefit it is that remedy which has shown itself capable of producing symptoms which most closely resemble those of the case demanding treatment—*e.g.* treat acute nephritis arising from scarlet fever with cantharides, which can produce (in large doses) acute nephritis. This principle is summed up in the general recommendation, "Let likes be treated with likes," which is not equivalent, as Dr. Heaney seems to think, to "Let identicals be treated with identicals." Now this recommendation, *similia similibus curantur*, was deduced from experience and is confirmed for those who hold to it by their daily practice. It is pre-eminently a matter that lends itself to experiment, and we physicians claim to be scientific men. Therefore I deprecate question-begging phrases like "discredited generalisation." Patient and careful investigation will enable any physician to have a basis of actual experience for his opinion, and I submit that only then does it become really a valuable contribution to the discussion.

Yours faithfully,

C. E. Wheeler.

